# The Assessment of the Osteoporosis Self-Assessment Tool for Asians and Calcaneal Quantitative Ultrasound in Identifying Osteoporotic Fractures and Falls Among Chinese People

**DOI:** 10.3389/fendo.2021.684334

**Published:** 2021-05-10

**Authors:** Chao Gao, Huijiang Song, Bihua Chen, Zhenlin Zhang, Hua Yue

**Affiliations:** ^1^ Shanghai Clinical Research Center of Bone Diseases, Department of Osteoporosis and Bone Diseases, Shanghai Jiao Tong University Affiliated Sixth People’s Hospital, Shanghai, China; ^2^ Department of Internal Medicine, Caolu Community Health Service Center, Shanghai, China; ^3^ Department of Internal Medicine, Longhua Community Health Service Center, Shanghai, China

**Keywords:** osteoporosis self-assessment tool for Asians, calcaneus quantitative ultrasound, osteoporosis, osteoporotic fracture, falls

## Abstract

The lack of DXA has made the diagnosis and treatment of osteoporosis extremely difficult in the vast rural areas of China, which has the largest population with high risks of osteoporosis. The aims of this cross-sectional study were to evaluate the association between the osteoporosis self-assessment tool for Asians (OSTA) and calcaneus quantitative ultrasound (QUS) in populations residing in Shanghai, China, and their assessment in predicting osteoporotic fractures and falls. A population of 12,033 participants, including 1272 males (average age 68.3 ± 9.8 years, range 28–100 years) and 10,761 females (average 56.8 ± 11.4 years, range 23–99 years), was gathered. OSTA and calcaneus QUS (Sonost 2000, OsteoSys) values were measured. Spearman’s correlation and Cohen’s kappa were used to determine the association and agreement between the OSTA and QUS. Receiver operating characteristic (ROC) curves were adapted to assess the performance and optimal cutoff values for the OSTA and QUS in osteoporotic fracture and fall screening. In total, the prevalence of osteoporotic fractures (low-trauma fractures including fractures of the spine, hip, forearm, humerus and ribs) was 15.2% in women, and 17.7% reported a history of falls (falling from standing height more than once in the past year). The percentages of men with the same history were 8.4% and 11.7%, respectively. The association between the OSTA and QUS was found to be *r_s_* = 0.393, κ = 0.137, *p* < 0.001. The OSTA (cutoff < −1) revealed an area under ROC curve (AUC) of 0.590 in identifying female individuals with moderate or high risk of osteoporosis defined by QUS (T-score < −1). The QUS T-score lower than −1.55 or −1.40 in postmenopausal women may lead to an increased risk of falls or osteoporotic fractures, respectively. The agreement between QUS and the OSTA seemed to be limited in determining individuals at risk of osteoporosis. Measuring bone mineral density (BMD) by dual energy X-ray absorptiometry (DXA) may still be necessary in the clinical diagnosis of osteoporosis. OSTA and QUS T-scores less than the respective cutoff values may indicate an increased risk of osteoporotic fractures and falls that individual should be further treated and screened by DXA.

## Introduction

Osteoporosis is a bone disease characterized by low bone mass, compromised bone strength and deterioration of the bone architecture, resulting in an increased risk of falls and fractures (1). Bone mass and strength include both bone density and bone quality (2). China has the largest population in the world, and the prevalence of osteoporosis has also escalated in recent years due to the rapid aging of the population, which has become a major public health problem. The National Health Commission of the People’s Republic of China revealed the results of the first epidemiological survey of osteoporosis in China in October 2018. The results showed that the prevalence of osteoporosis was 19.2% in the group over 50 years old, while it increased to 32.0% in the group over 65 years old (3). On the other hand, the prevalence is higher in rural areas of China, at 20.7% and 35.3%, respectively, which may indicate a relatively poor bone health situation of countryside inhabitants. Our former study concentrated on vertebral fractures in approximately 15,000 Shanghai community-dwelling elderly individuals and found a prevalence of 17.0% for males and 17.3% for females, and rural areas also obtained a higher prevalence than downtown areas in Shanghai (4). Bone mineral density (BMD) measured by dual energy X-ray absorptiometry (DXA) remains the gold diagnostic standard for osteoporosis according to a report about the assessment of fracture risk and its application to screening for postmenopausal osteoporosis from the World Health Organization in 1994. However, DXA is only available in large general hospitals in China and limited by its inconvenience, professional operation and high cost; it may not be suitable for wide screening of osteoporosis (5, 6), especially in extensive rural areas of China.

Quantitative ultrasound (QUS) is a noninvasive method developed to evaluate skeletal microarchitectures and bone mass except BMD (7). It also has outstanding portability and operability (8). The detection sites of QUS include the calcaneus, phalanx and wrist. The calcaneus is the most valuable site for diagnosis because cancellous bone in the calcaneus can reflect the bone mineral density and trabecular microstructure information more accurately than cortical bone in other sites (9). And QUS also has other ultrasound technique that might quantify the bone mineral density such as the radius low-frequency axial ultrasound velocity (10) and the Radiofrequency echographic multi-spectrometry (REMS) (11) which provide reliable approaches for improved bone strength and fracture risk estimations. These are also some screening tools adapted to predict the risk of osteoporosis and osteoporotic fracture, such as the fracture risk assessment tool (FRAX), osteoporosis self-assessment tool (OST), and simple calculated risk estimation (SCORE) (12, 13). FRAX is a powerful screening tool that obtains the ability of identifying patients at high risk of fractures with or without BMD and the latest therapeutic options may provide new way to solve critical issues in the management of osteoporosis and related fractures (14, 15). But most of these tools were validated and carried out in the Caucasian population and without BMD, FRAX is only available in examples be the well women at menopause with no clinical risk factors. Koh et al. (16) suggested the osteoporosis self-assessment tool for Asians (OSTA) in 2001 based on the age and body weight of Asian women. The performance of the OSTA has also been subsequently validated against DXA in Chinese women and men (17–19). Diem et al. also indicted the OSTA perform better in men older than 70 years for osteoporosis screening than FRAX (20). OSTA and QUS, respectively, and their combination had already be validated to be helpful in finding populations at high risk for osteoporosis, which could be an alternative method for diagnosing osteoporosis in Chinese postmenopausal women and elderly men, especially in areas where DXA measurement is not accessible (21, 22). The International Osteoporosis Foundation (IOF) One Minute Osteoporosis Risk Assessment Test (23) is also a feasible way to check whether one individual is at risk of osteoporosis and fractures.

The prevalence of osteoporosis is much higher in rural areas of China than in downtown areas (3). However, the lack of DXA has made the diagnosis and treatment of osteoporosis extremely difficult in the vast rural areas of China, which has the largest population with a high risk of osteoporosis. Few studies concentrate on the agreement between the OSTA and QUS in identifying the risk of osteoporosis or history of fractures and falls in Chinese people. Our study aims to determine the association between the OSTA and QUS in populations residing in rural or community areas in Shanghai, China, and to assess the identification of osteoporotic fractures or falls. If specific agreement between the two screening tools is found, it may provide a simple, inexpensive and effective screening method for millions of Chinese rural and grassroots populations with relatively low levels of medical care to screen for the risk of osteoporosis.

## Methods

### Study Population and Protocol

This cross-sectional study was conducted from March 2019 to July 2019. The participants were gathered voluntarily in two communities in Shanghai. Anyone in communities without a history or evidence of metabolic bone diseases such as osteomalacia, osteogenesis imperfecta or Paget’s disease who was able to read and provide informed consent was included. All subjects were contacted by the staff or family doctors in the Community Health Service Center where they lived and invited to the Community Health Service Center for measurements of calcaneal ultrasound by QUS. Then, further interview was conducted by family doctors for the following information: demographic information; history of low-trauma fracture, including fractures of the spine, hip, forearm, humerus and ribs, except fracture caused by traffic accidents or severe trauma; history of falls in the IOF One Minute Osteoporosis Risk Assessment Test (falling from standing height more than once in the past year; falls from high level were excluded); history of related metabolic bone diseases; and age at menopause in women. Their age was determined by their identification cards, height was measured using a height stadiometer, and weight was measured in light clothing without shoes by a weighing scale. The protocol of the study was approved by the Ethics Committee of Human Research of the Shanghai Jiao Tong University Affiliated Sixth People’s Hospital.

### Ultrasound Bone Densitometer and OSTA Index

A Sonost 2000 ultrasound bone densitometer (OsteoSys, Seoul, Korea) was adapted to measure the T-score of the left heel according to the operations manual provided by the manufacturer. The T-score is calculated as standard deviations from the average performance in young adults and output by the ultrasound device directly. Decreased values of bone ultrasound were defined as T-score results below −1. We adapted the same bone health status stratification by the WHO based on the DXA T-score as T-score > −1 as low risk, T-score ≤ −2.5 as high risk, and intermediate values as moderate risk because we believe that there is similarity between the two methods for risk assessment for fractures. The reduction of 1 standard deviation in the parameters of bone ultrasound is associated with increased risk, similar to the reduction in DXA bone densitometry. Meanwhile, the OSTA was calculated based on the formula: 0.2× (body weight − age), where the decimals of the product were truncated to yield an integer, and the participants were also assigned to respective risk groups based on the OSTA score: low risk (OSTA > −1), medium risk (−4 ~ −1), or high risk (OSTA < −4) (16).

### Statistical Analysis

Statistical analyses were performed by SPSS version 22.0 (IBM, NY, USA). Normality of the data was determined using the Kolmogorov-Smirnov test. Receiver operating characteristic (ROC) curves were constructed for the OSTA index using the QUS T-score as the reference, and we also calculated the area under the curve of ROC curves to judge the value of the OSTA and QUS in predicting falls and fracture history. An AUC value of 0.5 or lower indicates an inability to identify the related risk of osteoporosis, while a value higher than 0.5 suggests potential predictability of the tool. Sensitivity and specificity were calculated, and subanalysis according to age group, sex, and BMI was also performed. Alternative cutoff values of the OSTA and T-score were obtained by coordinate tracing of the ROC curve. The optimal cutoff value should obtain the highest Youden index and reasonable sensitivity and specificity. Correlations between the QUS T-score and OSTA index were determined by Spearman’s correlation, and Cohen’s kappa statistics were adapted for agreement analysis. A correlation coefficient between 0.5 and 1 is considered as high degree of correlation, while a correlation coefficient between 0.3 and 0.49 is considered as moderate degree of correlation. A kappa value (κ) of >0.6 was considered moderate, while >0.8 was strong. *p <*0.05 was considered statistically significant (two-tailed).

## Results

A population of 13,505 participants was gathered and finally, 12,033 participants, including 1272 males (average age 68.3 ± 9.8 years, range 28–100 years) and 10,761 females (average 56.8 ± 11.4 years, range 23–99 years) accepted the interview by family doctors from March 2019 to July 2019. The characteristics of the men and women are shown in **Table 1**. Approximately 15.2% (1636/10761) of women suffered a history of low-trauma fracture in their lifetime (including fractures of the spine, hip, forearm, humerus and ribs; fractures caused by traffic accident or severe trauma were excluded), and 17.7% (1907/10761) reported a history of falls more than once in the last year (falling from standing height without force; falls from a high level were excluded). The percentages of men with the same history were 8.4% (107/1272) and 11.7% (149/1272), respectively. The prevalences of T-scores > −1, intermediate values and T-scores ≤ −2.5 were 36.0%, 56.3%, and 7.7% for women and 26.7, 65.3 and 8.0% for men, respectively. Based on the OSTA, in women, 71.6% had low risk, 23.8% had moderate risk, and 4.6% had high risk of osteoporosis, while it was 65.0, 27.3 and 7.7% for men, respectively. The general agreement and correlation between the OSTA and QUS are shown in **Table 2** and were *κ* = 0.137 and *r_s_* = 0.393 (*p* < 0.001) based on sex classification, respectively. Further analysis based on sex showed that the kappa agreement between the OSTA and QUS was good in women (*κ* = 0.151, *p* < 0.001) but poor in men (*κ* = 0.059, *p* = 0.516). In terms of correlation, subanalysis based on sex between the QUS T-score and OSTA score was better in women (*r_s_* = 0.418, *p* < 0.001) than in men (*r_s_* = 0.144, *p* < 0.001).

**Table 1:** Characteristics of the 12033 study population in Shanghai, China.

Female (n=10761)								
Age (yr)	n	Height (cm)	Weight (kg)	BMI (kg/m^2^)	OSTA index	QUS *T*-score	History of fractures (%)	History of falls (%)
−49	2658	159.32 ± 4.91	59.59 ± 8.78	23.47 ± 3.26	3.68 ± 2.09	−0.59 ± 0.95	7.5	9.4
50–59	3190	158.01 ± 5.21	61.14 ± 8.48	24.48 ± 3.15	1.24 ± 1.78	−1.10 ± 0.93	14.2	14.8
60–69	3725	157.39 ± 5.27	60.86 ± 8.45	24.57 ± 3.27	−0.63 ± 1.79	−1.54 ± 0.84	19.8	23.3
70–79	1013	155.80 ± 5.64	58.86 ± 8.90	24.25 ± 3.49	−2.83 ± 1.92	−1.76 ± 0.92	20.6	26.0
80-	175	154.06 ± 5.97	55.50 ± 9.06	23.37 ± 3.50	−5.83 ± 1.97	−2.11 ± 0.93	21.1	30.3
**Male (n=1272)**								
−49	49	168.14 ± 6.95	70.22 ± 10.93	24.80 ± 3.27	5.58 ± 5.48*	−1.07 ± 0.87*	/	/
50–59	139	170.36 ± 6.50	71.67 ± 9.38	24.69 ± 2.96	3.28 ± 1.89*	−1.17 ± 0.96*	7.9*	8.6*
60–69	505	170.35 ± 5.46	70.12 ± 9.31	24.15 ± 2.98	0.96 ± 1.91*	−1.38 ± 0.89*	8.5*	12.1*
70–79	462	167.59 ± 5.55	68.13 ± 9.12	24.24 ± 2.95	−1.16 ± 1.99*	−1.41 ± 0.92*	7.1*	10.8*
80-	117	167.61 ± 6.10	65.80 ± 9.78	23.42 ± 3.32	−3.85 ± 2.16*	−1.55 ± 0.86*	10.3*	17.1*

BMI, body mass index; OSTA, osteoporosis self-assessment tool for Asians; QUS, quantitative ultrasound.

*P < 0.001 vs. women.

**Table 2:** The agreement and correlation between OSTA and QUS.

	*κ*	*p*	*r_s_*	*p*
Women	0.151	**< 0.001**	0.418	**< 0.001**
Men	0.011	0.516	0.144	**< 0.001**
Overall	0.137	**< 0.001**	0.393	**< 0.001**

OSTA, osteoporosis self-assessment tool for Asians; QUS, Quantitative ultrasound; κ, kappa coefficient; r_s_, Spearmen’s correlation coefficient. Significant p values are bolded.

ROC curves were adapted to determine the performance of the OSTA against QUS. The assessment of the OSTA (cutoff < −1) to identify individuals with moderate or high risk of osteoporosis defined by QUS (*T*-score < −1) was statistically significant in women (sensitivity = 44.3%, specificity =73.0%, AUC =0.586; 95% CI: 0.527–0.601; *p*<0.001), while no predictability was found in men (sensitivity =34.6%, specificity =70.9%, AUC =0.528; 95% CI: 0.493–0.562; *p*=0.116). At the cutoff < −4, the OSTA performed similarly in identifying individuals with a high risk of osteoporosis defined by QUS (*T*-score ≤ −2.5) in women (sensitivity =20.9%, specificity =96.2%, AUC =0.585; 95% CI: 0.563–0.608; *p*<0.001) and men (sensitivity =12.7%, specificity =92.7%, AUC =0.527; 95% CI: 0.467–0.588; *p*=0.358). The OSTA showed relatively higher sensitivity (women: 44.3% *vs.* 20.9%; men 34.6% *vs.* 12.7%) and lower specificity (women: 73.0% *vs.* 96.2%; men 70.9% *vs.* 92.7%) in identifying individuals with moderate or high risk of osteoporosis compared with high risk of osteoporosis (**Tables 3**, **4**) at the cutoff values of < −1 than of < −4. Further analysis based on age groups and BMI was also adapted in both women and men. The performance of the OSTA (cutoff < −1) in identifying individuals with moderate or high risk of osteoporosis was better among women (AUC = 0.586) than men (AUC = 0.528). A similar result was observed in identifying subjects with high risk of osteoporosis at a cutoff < −4 in women (AUC = 0.585) and men (AUC = 0.527). Subanalysis focused on the OSTA (cutoff < −1) identifying individuals with moderate or high risk of osteoporosis (T-score < −1) in different age groups showed that the sensitivity increased with aging for both sexes (women: 2.0% to 99.3%; men: 0.0% to 90.0%). Compared with men aged 79 or younger, the performance of the OSTA in identifying moderate or high risk of osteoporosis was better in women (sensitivity = 0.0%–51.5%, AUC = 0.496–0.531 *vs.* sensitivity = 2.0%–84.0%, AUC = 0.501–0.552, respectively), and the same trend was found for identifying subjects with high risk of osteoporosis at a cutoff < −4 for the OSTA.

**Table 3:** The performance of OSTA in identifying individuals with moderate or high risk of osteoporosis (QUS *T* score < − 1).

OSTA < −1 *vs T* score < −1												
	Female						Male					
	n	Sen.	Spe.	AUC	95% CI	*p*	n	Sen.	Spe.	AUC	95% CI	*p*
Overall	10761	44.3%	73.0%	0.590	0.527–0.601	**<0.001**	1272	34.6%	70.9%	0.528	0.493–0.562	0.116
Age												
−49	2658	2.0%	100.0%	0.501	0.447–0.524	0.945	49	0.0%	100.0%	0.500	0.331–0.669	1.000
50–59	3190	10.3%	94.6%	0.524	0.504–0.544	**0.018**	139	1.1%	98.0%	0.496	0.396–0.596	0.936
60–69	3725	43.3%	67.1%	0.552	0.530–0.573	**<0.001**	505	16.2%	89.9%	0.531	0.476–0.585	0.297
70–79	1013	84.0%	23.8%	0.539	0.493–0.584	0.089	462	51.5%	47.8%	0.497	0.439–0.554	0.909
80-	175	99.3%	0.0%	0.497	0.375–0.619	0.957	117	90.0%	11.1%	0.506	0.380–0.631	0.930
BMI												
Underwight	239	80.7%	53.8%	0.673	0.597–0.749	**<0.001**	34	96.3%	0.0%	0.481	0.243–0.720	0.881
Normal	6547	45.5%	83.4%	0.644	0.631–0.657	**<0.001**	776	44.6%	60.8%	0.527	0.483–0.572	0.235
Overweight	3975	13.3%	95.0%	0.542	0.524–0.560	**<0.001**	462	11.8%	89.9%	0.508	0.452–0.564	0.775

AUC, area under curve; CI, confidence interval; Sen., sensitivity; Spe., specificity. Significant p values (p<0.05) are bolded.

**Table 4:** The performance of OSTA in identifying individuals with high risk of osteoporosis (QUS *T* score < − 2.5).

OSTA < −4 *vs T* score < −2.5										
	Female						Male			
	Sen.	Spe.	AUC	95% CI	*p*	Sen.	Spe.	AUC	95% CI	*p*
Overall	20.9%	96.2%	0.585	0.563–0.608	**<0.001**	12.7%	92.7%	0.527	0.467–0.588	0.358
Age										
−49	0.0%	100.0%	0.500	0.396–0.604	1.000	\	\	\	\	\
50–59	8.0%	99.9%	0.504	0.450–0.557	0.890	0.0%	100.0%	0.500	0.280–0.720	1.000
60–69	5.6%	97.6%	0.516	0.485–0.547	0.296	0.0%	99.2%	0.469	0.395–0.597	0.935
70–79	42.7%	73.7%	0.582	0.583–0.626	**<0.001**	6.7%	91.6%	0.491	0.404–0.579	0.849
80-	83.8%	15.0%	0.494	0.406–0.582	0.892	58.8%	54.0%	0.564	0.417–0.711	0.399
BMI										
Underwight	65.0%	61.8%	0.634	0.540–0.728	**0.007**	50.0%	73.3%	0.617	0.307–0.926	0.454
Normal	24.0%	95.4%	0.597	0.569–0.624	**<0.001**	16.2%	91.1%	0.536	0.462–0.611	0.321
Overweight	5.3%	99.2%	0.528	0.488–0.567	0.151	0.0%	100.0%	0.500	0.393–0.607	1.000

AUC, area under curve; CI, confidence interval; Sen., sensitivity; Spe., specificity. Significant p values (p<0.05) are bolded.

Based on BMI, the OSTA (cutoff < −1) showed a good performance in predicting underweight, normal and overweight individuals with moderate or high risk of osteoporosis (sensitivity = 13.3%–80.7%, specificity = 53.8%–95.0%, AUC = 0.542–0.673, *p*< 0.001), and the OSTA (cutoff < −4) also had predictability in underweight and normal individuals with high risk of osteoporosis (sensitivity = 65.0%, 24.0%; specificity = 61.8%, 95.4%; AUC = 0.634, 0.597; *p*= 0.007, *p*< 0.001, respectively) among women. The sensitivity of the OSTA (cutoff < −1) in predicting normal weight men with moderate or high risk of osteoporosis (sensitivity = 44.6%) was better than at the cutoff < −4 (sensitivity = 16.2%). A similar trend was observed for the identification of overweight men with moderate or high risk of osteoporosis at a cutoff < −1 (sensitivity = 11.8%) and high risk of osteoporosis at a cutoff < −4 (sensitivity = 0%) (**Tables 3**, **4**).

**Figure 1:**
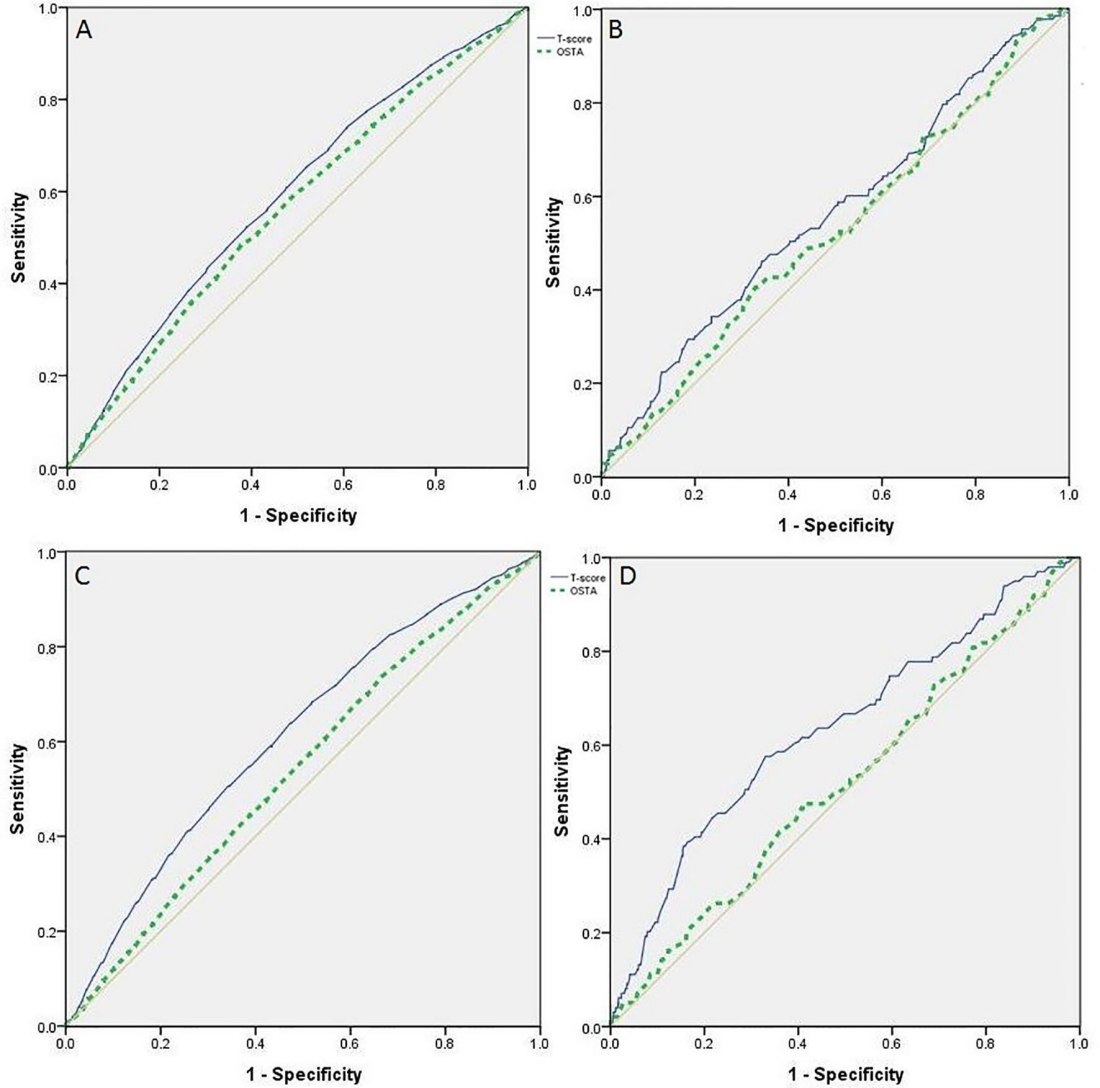
ROC curves of OSTA index and QUS T-score in identifying the risk of fall down and fracture, postmenopausal women, men over 50 years old. **(A)** ROC on the performance of OSTA and QUS T-score in identifying postmenopausal women at risk of fall down. **(B)** ROC on the performance of OSTA and QUS T-score in identifying men over 50 years old at risk of fall down. **(C)** ROC on the performance of OSTA and QUS T-score in identifying postmenopausal women at risk of fracture. **(D)** ROC on the performance of OSTA and QUS T-score in identifying men over 50 years old at risk of fracture.

ROC curves were also adapted to evaluate the performance of QUS and the OSTA in screening the risk of osteoporotic fractures and falls in postmenopausal women and men over 50 years old (**Figure 1** **)**, and appropriate cutoff values with the highest Youden index were chosen. The OSTA and QUS T-score showed certain ability in predicting falls (cutoff value= −0.40; sensitivity =48.3%, specificity =62.3%, AUC =0.565; 95% CI: 0.550–0.579; Youden index=0.106; *p*<0.001 and cutoff value= −1.55; sensitivity =52.1%, specificity =61.3%, AUC =0.592; 95% CI: 0.577–0.606; Youden index=0.134; *p*<0.001, respectively) and fragility fracture (cutoff value= 1.40; sensitivity =73.6%, specificity =33.6%, AUC =0.543; 95% CI: 0.527–0.558; Youden index=0.072; *p*<0.001 and cutoff value= −1.40; sensitivity =63.8%, specificity =52.8%, AUC =0.612; 95% CI: 0.597–0.627; Youden index=0.166; *p*<0.001, respectively) among postmenopausal women. The performance of the OSTA and QUS T-score in identifying the risk of fragility fractures and falls was relatively poor among men over 50 years old with lower sensitivity, AUC and Youden index than women except for QUS T-score *vs.* fracture (cutoff value= −1.80; sensitivity =57.6%, specificity =67.0%, AUC =0.634; 95% CI: 0.574–0.695; Youden index=0.246; *p*<0.001) (**Table 5**). We also determined the performance of combining the OSTA and QUS *T*-score in predicting the risk of fragility fracture and fall history, and a new regression curve variable (*Y*) based on the OSTA index and QUS-T score was fitted as *Y* = −0.389QUS - 0.002OSTA - 2.191 and *Y=* −0.275QUS - 0.048OSTA - 1.834 for postmenopausal women, and *Y=* −0.566QUS - 0.018OSTA - 3.317 and *Y=* −0.262QUS - 0.033OSTA – 2.412 for men over 50 years old. Higher values than the optimal cutoff point of *Y* represent potential high risk. A comparison of AUCs was also made between *Y* and the OSTA index or QUS-T score alone for detecting subjects with fractures or falls; however, no significant difference was seen in men and women. On the other hand, neither sensitivity nor specificity was optimized in identifying fractures or falls by combining the OSTA with the QUS-T score.

**Table 5:** Optimal cut-off values according to gender group in identifying individuals at risk of fall down and fracture.

	Postmenopausal women (n= 9031 in total) (n= 1531 for fractures, = 1762 for falls)							Men over 50 (n= 1223 in total) (n= 99 for fractures, = 143 for falls)						
	Cutoff value	Sen.	Spe.	AUC	95% CI	Youden index	*p*	Cutoff value	Sen.	Spe.	AUC	95% CI	Youden index	*p*
OSTA *vs* Fall down	−0.35	48.3%	62.3%	0.565	0.550–0.579	0.106	**<0.001**	−1.20	40.6%	67.0%	0.562	0.511–0.613	0.076	**0.015**
T score *vs* Fall down	−1.55	52.1%	61.3%	0.592	0.577–0.606	0.134	**<0.001**	−1.75	46.2%	65.6%	0.523	0.472–0.574	0.118	0.365
OSTA-T score *vs* Fall down	−1.50	62.3%	52.7%	0.597	0.583–0.611	0.150	**<0.001**	−2.00	52.4%	59.4%	0.564	0.514–0.615	0.118	**0.012**
OSTA *vs* Fracture	1.40	73.6%	33.6%	0.543	0.527–0.558	0.072	**<0.001**	−0.45	47.5%	59.1%	0.520	0.460–0.580	0.066	0.502
T score *vs* Fracture	−1.40	63.8%	52.8%	0.612	0.597–0.627	0.166	**<0.001**	−1.80	57.6%	67.0%	0.634	0.574–0.695	0.246	**<0.001**
OSTA-T score *vs* Fracture	−1.65	63.4%	53.6%	0.612	0.597–0.627	0.170	**<0.001**	−2.10	40.4%	83.3%	0.635	0.574–0.695	0.237	**<0.001**

The table indicates the optimal cut-off values for OSTA or QUS T-score based on results of ROC curve for each gender group. Cut-off values with highest Youden Index were selected.

AUC, area under curve; CI, confidence interval; Sen., sensitivity; Spe., specificity. Significant p (p<0.05) values are bolded.

## Discussion

The results showed that the agreement between the OSTA and QUS was limited despite a statistically significant correlation, and this correlation was better in women than in men. Chen et al. (24) conducted a study in 553 individuals and found a similar correlation in Taiwanese men (*r* = 0.50) and women (*r* = 0.54). Further analysis revealed that the performance of the OSTA was mediocre for women but unpredictive for men in identifying subjects at risk of osteoporosis defined by QUS. This was in accordance with the study conducted by Subramaniam et al. (25) with DXA and Chin et al. (26) with QUS in Malaysians. The OSTA index or QUS-T score may be more adaptable for clinically detecting the risk of osteoporosis in female patients.

ROC analysis was also adapted to detect the performance of the OSTA in identifying individuals with moderate and high risk (QUS T-score < −1) of osteoporosis or high risk (QUS T-score < −4) of osteoporosis. The AUC values were over 0.5, but the sensitivity was low in women. This result does not agree with some previous studies with high AUC and sensitivity values for the OSTA in identifying individuals with osteoporosis defined by the DXA T-score (27, 28). Studies conducted in Nepali (8) (n=100, mean age =58.1 years) and Malaysian (26) (n=362, mean age =61.7 years) women showed that the OSTA score demonstrated sensitivity values of 85.2% and 54.8% at the cutoff of < −1 in identifying individuals with a QUS T-score of < −1. These values were higher than the sensitivity of 44.3% in our study of women (n=10,761, mean age =56.8 years). This result may be because the individuals in our study have a larger age range (28–100 years for men and 23–99 years for women), and many kinds of screening tools for identifying osteoporosis risk, including the OSTA, were found to have better performance in postmenopausal women and elderly men (29, 30). It also suggested that the OSTA and QUS were not suitable in osteoporosis risk screening in premenopausal women and younger men, and their agreement was relatively weak in the total population at all age groups.

QUS and the OSTA have important clinical roles in screening patients with a high risk of osteoporosis and osteoporotic fracture. Many osteoporotic fractures at the distal forearm, vertebral bodies and hip are closely related to falls (31). The cutoff points for men are relatively lower than those for women mainly because bone mineral density is higher in men than in women. Many other studies focusing on the cutoff points of QUS T-scores in identifying osteoporotic fractures also found similar cutoff points as our studies. Liu et al. (32) set up a study focused on the associations between calcaneus QUS and clinical vertebral fractures and nonvertebral fractures in 9325 Chinese people found very close cutoff points to our results for detecting osteoporotic fractures, including spine, hip, forearm, humerus and ribs in postmenopausal women and men over 50 years old. Although the model of calcaneus QUS parameters was not the same in these two studies (GE Lunar Corp., Madison, WI *vs.* OsteoSys, Seoul, Korea), a similar population in Shanghai, China, led to the approximate result. Other research investigated much lower cutoff-off points than our study. For example, QUS T-scores of −2.5 and −2.2 were suggested to predict hip fracture risk in two studies conducted in Swiss women (33, 34). Compared with those two studies conducted in Shanghai, two studies in Switzerland obtained a relatively older population, and different fracture sites and races may also contribute to lower results. The OSTA is the first osteoporosis screening method for women catering to Asian populations established by a multinational Asian cohort, and it has also been expanded to identify osteoporosis in men and to determine fracture risk (35). The OSTA seemed to be capable of screening fracture resulting from low-energy trauma in postmenopausal women in China (36) but the low AUC result may suggest the poor ability of the OSTA in identifying fractures in Chinese postmenopausal women, which was worse in men over 50 in our study (AUC=0.520, 95% CI: 0.460–0.580).Meanwhile, the result of AUC < 0.8 may indicate a poor ability of the OSTA to predict a fall in Chinese people which is similar to other study (24). On the other hand, many previous studies focused on the relationship between QUS and history or risk of falls, proving that there are several advantages of QUS as a viable alternative to DXA for assessing osteoporotic hip fracture and fall risk (37, 38). Calcaneus QUS devices have also been proven to be as accurate in predicting clinical osteoporosis fractures in elderly women as BMD by DXA (39–41), but the ability of QUS to predict osteoporotic fractures is not widely applied.

The regression curves were fitted to present the performance when combining the OSTA and QUS in osteoporotic fracture and fall screening. The area under the ROC curve drawn by the predicted value with a history of fractures and falls together with the Youden index was calculated. Higher Youden indices were obtained when the OSTA was combined with QUS in osteoporotic fracture and fall screening, indicating a better and more truthful test. Some researchers noticed that using the OSTA or QUS alone as the single standard of screening osteoporosis may cause low sensitivity, specificity and Youden index that lead to missed and delayed diagnosis (42, 43). We adapted regression curves to reach the predictive value *Y* and a regression equation for combining the OSTA and QUS in predicting osteoporotic fracture and fall. Any *Y* indices higher than the predictive value may indicate an increase in risk. When we combined the OSTA and QUS in predicting falls, a higher sensitivity and a higher Youden index were found, which were the opposite in predicting osteoporotic fractures in postmenopausal women and men over 50 years old. This may lead to the result that combined screening had better performance in predicting falls, while QUS alone is more reasonable in identifying osteoporotic fractures.

Our study was also subject to a number of limitations. First, the participants in our study did not undergo the DXA exam at the time of the study, and the prevalence of osteoporosis based on BMD was not determined. Therefore, the ability of the OSTA and QUS to identify the risk of osteoporosis defined by BMD could not be determined, and agreement between DXA, QUS and the OSTA was not established in this study. Second, many other QUS data, such as broadband ultrasound attenuation, stiffness index and speed of sound, were not recorded in the study, and all the fracture and fall histories recorded in the study were self-reported by participants, so there was a possibility of missing reports. The relatively low agreement between the OSTA and QUS classification could be due to several reasons. The cutoff point of the OSTA in identifying osteoporotic individuals was established according to BMD values measured by DXA (16, 19). QUS indices were significantly correlated with BMD (44, 45). However, soft tissues and edema at the heel can artificially attenuate the transmission of ultrasound across the calcaneus, and indices of QUS are influenced by skeletal microstructures and bone strength, which are not reflected in BMD (7). These factors could weaken the agreement between QUS and the OSTA.

In conclusion, although the agreement between QUS and the OSTA was not so significant, their abilities were limited in determining individuals at risk of osteoporosis, including fractures and falls. Combining QUS and the OSTA in screening may improve their ability to predict osteoporotic fractures and falls. We suggest that a QUS T-score measured from an OsteoSys QUS device for a postmenopausal woman or man over 50 years old of less than −1.40 and −1.80, respectively, may indicate an increased risk of osteoporotic fractures and should signal further central DXA examinations. Measuring bone mineral density by DXA may still be necessary in the clinical diagnosis of osteoporosis, but QUS still has several advantages over DXA as a clinical case-finding strategy, and the OSTA is a simple, totally noninvasive and inexpensive osteoporosis risk screening tool. Its lower cost, portability, and lack of ionizing radiation present greater advantages in the clinical screening and diagnosis of osteoporosis in the developing world (46), especially in many unenlightened regions in China. Further studies may be carried out to validate the details and results.

## Data Availability Statement

The original contributions presented in the study are included in the article/supplementary material. Further inquiries can be directed to the corresponding author.

## Ethics Statement

The studies involving human participants were reviewed and approved by the ethics committee of the Human Research of the Shanghai Jiao Tong University Affiliated Sixth People’s Hospital. The patients/participants provided their written informed consent to participate in this study.

## Author Contributions

Study design: ZZ and HY. Study conduct: CG, HS, and BC. Data collection: CG and HS. Data analysis and interpretation: CG, ZZ, and HY. Manuscript draft: CG. Manuscript content revision: ZZ and HY. CG takes responsibility for the integrity of the data analysis. CG and HS contributed equally to this work. All authors contributed to the article and approved the submitted version.

## Funding

The study was supported by the Shanghai Municipal Key Clinical Specialty, National Natural Science Foundation of China (Young Scholars: 81900808) and the Clinical Science and Technology Innovation Project of Shanghai Shenkang Hospital Development Center (SHDC12018120).

## Conflict of Interest

The authors declare that the research was conducted in the absence of any commercial or financial relationships that could be construed as a potential conflict of interest.
